# Insect Attraction versus Plant Defense: Young Leaves High in Glucosinolates Stimulate Oviposition by a Specialist Herbivore despite Poor Larval Survival due to High Saponin Content

**DOI:** 10.1371/journal.pone.0095766

**Published:** 2014-04-21

**Authors:** Francisco R. Badenes-Perez, Jonathan Gershenzon, David G. Heckel

**Affiliations:** 1 Department of Entomology, Max Planck Institute for Chemical Ecology, Jena, Germany; 2 Instituto de Ciencias Agrarias, Consejo Superior de Investigaciones Científicas, Madrid, Spain; 3 Department of Biochemistry, Max Planck Institute for Chemical Ecology, Jena, Germany; University of Arkansas, United States of America

## Abstract

Glucosinolates are plant secondary metabolites used in plant defense. For insects specialized on Brassicaceae, such as the diamondback moth, *Plutella xylostella* L. (Lepidoptera: Plutellidae), glucosinolates act as “fingerprints” that are essential in host plant recognition. Some plants in the genus *Barbarea* (Brassicaceae) contain, besides glucosinolates, saponins that act as feeding deterrents for *P. xylostella* larvae, preventing their survival on the plant. Two-choice oviposition tests were conducted to study the preference of *P. xylostella* among *Barbarea* leaves of different size within the same plant. *P. xylostella* laid more eggs per leaf area on younger leaves compared to older ones. Higher concentrations of glucosinolates and saponins were found in younger leaves than in older ones. In 4-week-old plants, saponins were present in true leaves, while cotyledons contained little or no saponins. When analyzing the whole foliage of the plant, the content of glucosinolates and saponins also varied significantly in comparisons among plants that were 4, 8, and 12 weeks old. In *Barbarea* plants and leaves of different ages, there was a positive correlation between glucosinolate and saponin levels. This research shows that, in *Barbarea* plants, ontogenetical changes in glucosinolate and saponin content affect both attraction and resistance to *P. xylostella*. Co-occurrence of a high content of glucosinolates and saponins in the *Barbarea* leaves that are most valuable for the plant, but are also the most attractive to *P. xylostella*, provides protection against this specialist herbivore, which oviposition behavior on *Barbarea* seems to be an evolutionary mistake.

## Introduction

According to the optimal defense theory, the most valuable parts of a plant should also be the most protected against herbivores [1], [2]. Young leaves are supposed to be more valuable than older ones because they can make a higher contribution to the fitness of the plant as a result of having relatively higher photosynthetic potential [3]. In agreement with this theory, it has been found that, within a plant, different organs and leaves can contain different concentrations of defense compounds [4], [5]. This the case for glucosinolates, plant secondary metabolites used for defense and found mainly in plants of the order Brassicales [6], [7], which have been found in higher concentrations in younger compared to older leaves within the same plant [4], [8]–[11]. At the whole plant level, glucosinolate content also changes over time, but not in a linear manner [4], [8], [12]. Like glucosinolates, saponins are plant secondary metabolites used in plant defense [13]–[15]. With the exception of insects specialized on saponin-rich plants [16], saponins act as feeding-deterrents and are toxic [17]–[19]. We have not found any studies addressing changes in saponin content with leaf age in Brassicaceae, but saponin content in leaves has been shown to decrease with leaf age in American holly, *Ilex opaca* Aiton (Aquifoliaceae) [20]. Saponin content also changes over time at the whole plant level, often increasing with plant age, although decreases with plant age and seasonal fluctuations have also been recorded [21]–[23]. In the genus *Barbarea* R. Br. (Brassicaceae), the only one in Brassicaceae where saponins have been found so far [24]–[28], seasonal fluctuations in saponin content seem to occur as inferred by changes in resistance to the flea beetle *Phyllotreta nemorum* L. (Coleoptera: Chrysomelidae) [29], [30].

The diamondback moth, *Plutella xylostella* L. (Lepidoptera: Plutellidae), is an insect herbivore specialized on glucosinolate-containing crucifers [31]–[33]. Specialist insects like *P. xylostella* have evolved mechanisms to avoid the toxicity of glucosinolates, which are used in host plant recognition and act as feeding and oviposition stimulants [7], [33]–[38]. Larvae of *P. xylostella* cannot survive on some varieties and types of *B. vulgaris* despite these plants being highly preferred for oviposition by *P. xylostella* adults [24], [25], [28], [39]–[42]. This oviposition mistake of *P. xylostella* has been investigated in pest management to use *Barbarea* plants as a dead-end trap crop for *P. xylostella*, which is considered one of the most damaging insect pests of cruciferous crops throughout the world [28], [32], [40], [43]. Among the varieties and types of *B. vulgaris* on which *P. xylostella* cannot survive are *B. vulgaris* var. *variegata* and G-type (glabrous) *B. vulgaris* var. *arcuata*, while P-type (pubescent) *B. vulgaris* var. *arcuata* allows survival of *P. xylostella* larvae [24], [25]. The resistance of *B. vulgaris* to *P. xylostella* is caused by the triterpenoid saponins 3-*0*-*β*-cellobiosylhederagenin (saponin 1) and 3-*0*-*β*-cellobiosyloleanolic acid (saponin 2), which act as feeding-deterrents or are correlated with deterrency in *P. xylostella* larvae [24], [25]. Other *B. vulgaris* types and other *Barbarea* spp. containing saponins 1 and 2 are also resistant to *P. xylostella* [28].

Adult oviposition and larval feeding preference for younger over older leaves of a particular host plant is a common trend among many herbivorous insects, especially in specialists, including *P. xyllostella* [9], [44], [45]. Within the same plant, two-choice oviposition preference tests have shown that *P. xylostella* prefers to oviposit on younger leaves of <3.0 maximum leaf diameter compared to older leaves of >6.0 maximum leaf diameter of *B. vulgaris* [44]. Given the type of rosette growth of *Barbarea* plants, at the age of the plants used in the study by Badenes-Perez et al (2005) and here, leaf size was correlated with leaf age. Although containing lower content of toxic metabolites than younger leaves, older leaves may be less nutritious for insects than younger leaves [46], [47]. Feeding on older leaves can also increase insect mandibular wear more than feeding on younger leaves because of the increased toughness of older leaves [48]. At the whole plant level, the plant phenological age hypothesis also predicts that herbivores prefer and perform better on younger compared to older plants [49]. However, there are many cases in which insects prefer older plants over younger ones [50], [51]. Among *Brassica oleracea* L. and *B. vulgaris* plants aged between 6 and 14 weeks old, *P. xylostella* also preferred to oviposit on older versus younger plants, even though survival and development of *P. xylostella* larvae can be negatively affected by plant age [52].

In relation to the preference of *P. xylostella* for younger leaves and older plants of *B. vulgaris* [44], [52], it is not known whether there could be an association between this preference and plant concentrations of glucosinolates and saponins, the former being oviposition and feeding stimulants, and the latter preventing the survival of the insect on the plant. We hypothesize that, given the known attraction of *P. xylostella* to glucosinolates, if glucosinolate content in *Barbarea* leaves is higher in younger compared to older leaves as it happens in other Brassicaceae, *P. xylostella* would preferentially oviposit on young leaves. It is not known how saponins vary with leaf and plant age in *Barbarea* spp. and whether they are correlated with changes in glucosinolate content. A correlation between plant content of glucosinolates and saponins could protect *Barbarea* plants from specialist insects adapted to glucosinolates. Furthermore, as both glucosinolates and saponins are plant defense compounds, their co-occurrence would have implications for the protection of *Barbarea* plants, not only against *P. xylostella*, but against other herbivores as well. To test our hypotheses, we conducted two-choice oviposition preference tests and measured glucosinolate and saponin concentrations in *Barbarea* leaves of different size to test the association between leaf size, oviposition preference, and glucosinolate and saponin concentrations. We also measured glucosinolate and saponin content in plants of different age. Besides analyzing true leaves, we analyzed the glucosinolate and saponin content of cotyledons. Larval survival and oviposition preference tests were also conducted with cotyledons and true leaves within the same plant.

## Results

### Analysis of Glucosinolates and Saponins in Barbarea spp

**Figure 1:**
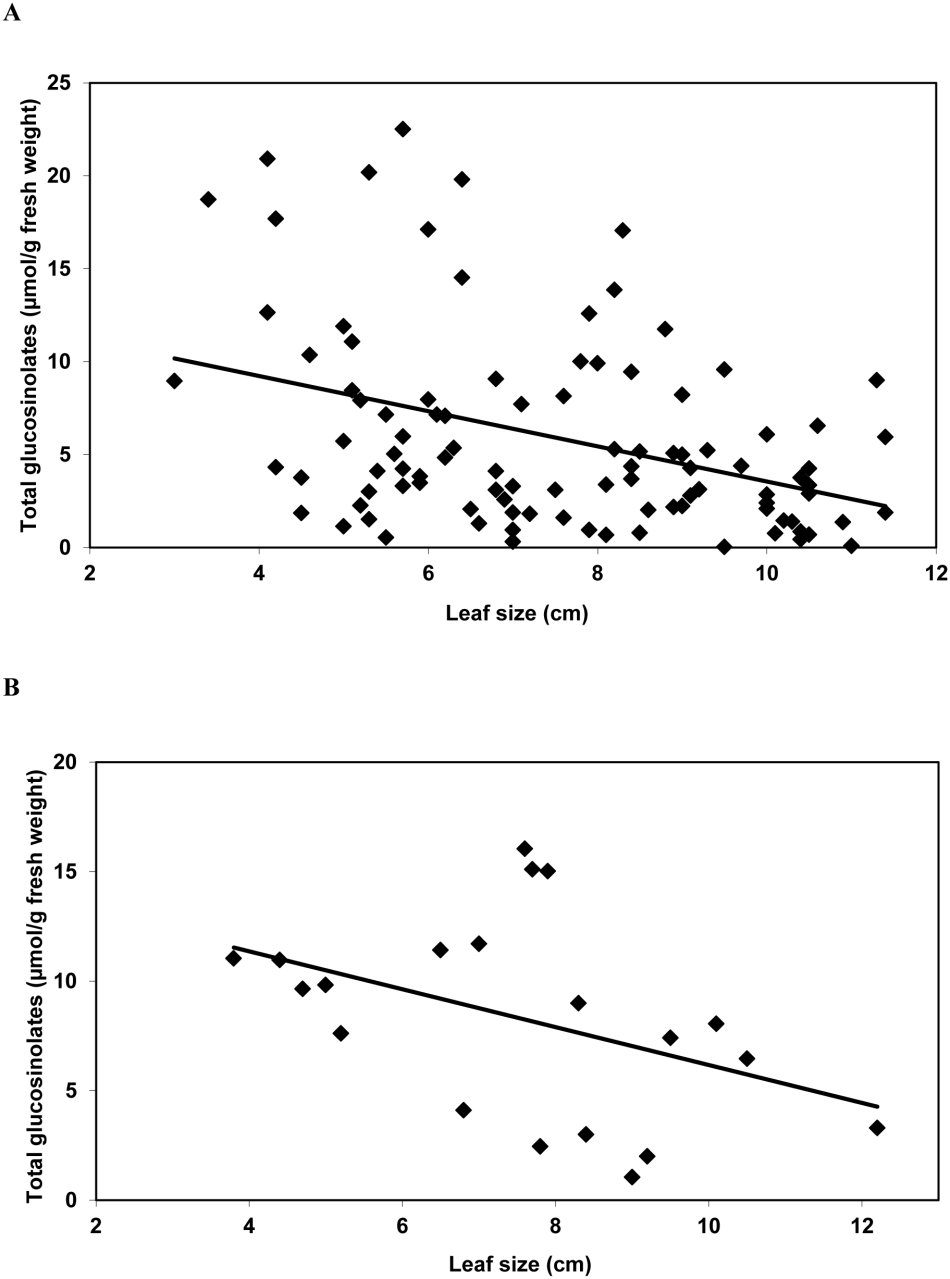
Total glucosinolates (µmol/g of plant fresh weight) in leaves of different size in G-type *Barbarea vulgaris* var. *arcuata* (A) and P-type *B.* *vulgaris* var. *arcuata* (B). For G- and P-type *B. vulgaris* leaves, maximum leaf width ranged from 3.0 to 11.4 cm (n = 100) and from 4.4 to 12.2 cm (n = 20), respectively.

A significant negative relationship was found between leaf size and content of glucosinolates for both G-type (*y* = 13.01–0.95*x*; *n* = 100; *r* = 0.39; *F*_1,98_ = 17.01; *P*≤0.001) and P-type *B. vulgaris* (*y* = 14.81–0.86*x*; *n* = 20; *r* = 0.44; *F*_1,19_ = 4.20; *P* = 0.050) (Figs. 1A and 1B). In G-type *B. vulgaris*, the glucosinolate that decreased the most with increasing leaf size was the dominant glucosinolate (*S*)-2-hydroxy-2-phenylethylglucosinolate (S2OH2PE) (*y* = 11.95–0.89*x*; *n* = 100; *r* = 0.38; *F*_1,98_ = 16.13; *P*≤0.001), but concentrations of (*R*)-2-hydroxy-2-phenylethylglucosinolate (R2OH2PE) (*y* = 0.26–0.17*x*; *n* = 100; *r* = 0.31; *F*_1,98_ = 10.34; *P* = 0.002), indol-3-ylmethylglucosinolate (I3M) (*y* = 0.64–0.04*x*; *n* = 100; *r* = 0.42; *F*_1,98_ = 21.01; *P*≤0.001), and 4-methoxyindol-3-ylmethylglucosinolate (4MOI3M) (*y* = 0.09–0.01*x*; *n* = 100; *r* = 0.29; *F*_1,98_ = 8.93; *P* = 0.003) also decreased with leaf size. In P-type *B. vulgaris*, only the dominant glucosinolate R2OH2PE (*y* = 4.25–1.07*x*; *n* = 20; *r* = 0.46; *F*_1,19_ = 4.69; *P* = 0.044) decreased significantly with leaf size; concentrations of the other glucosinolates found did not vary significantly with leaf size.

**Figure 2:**
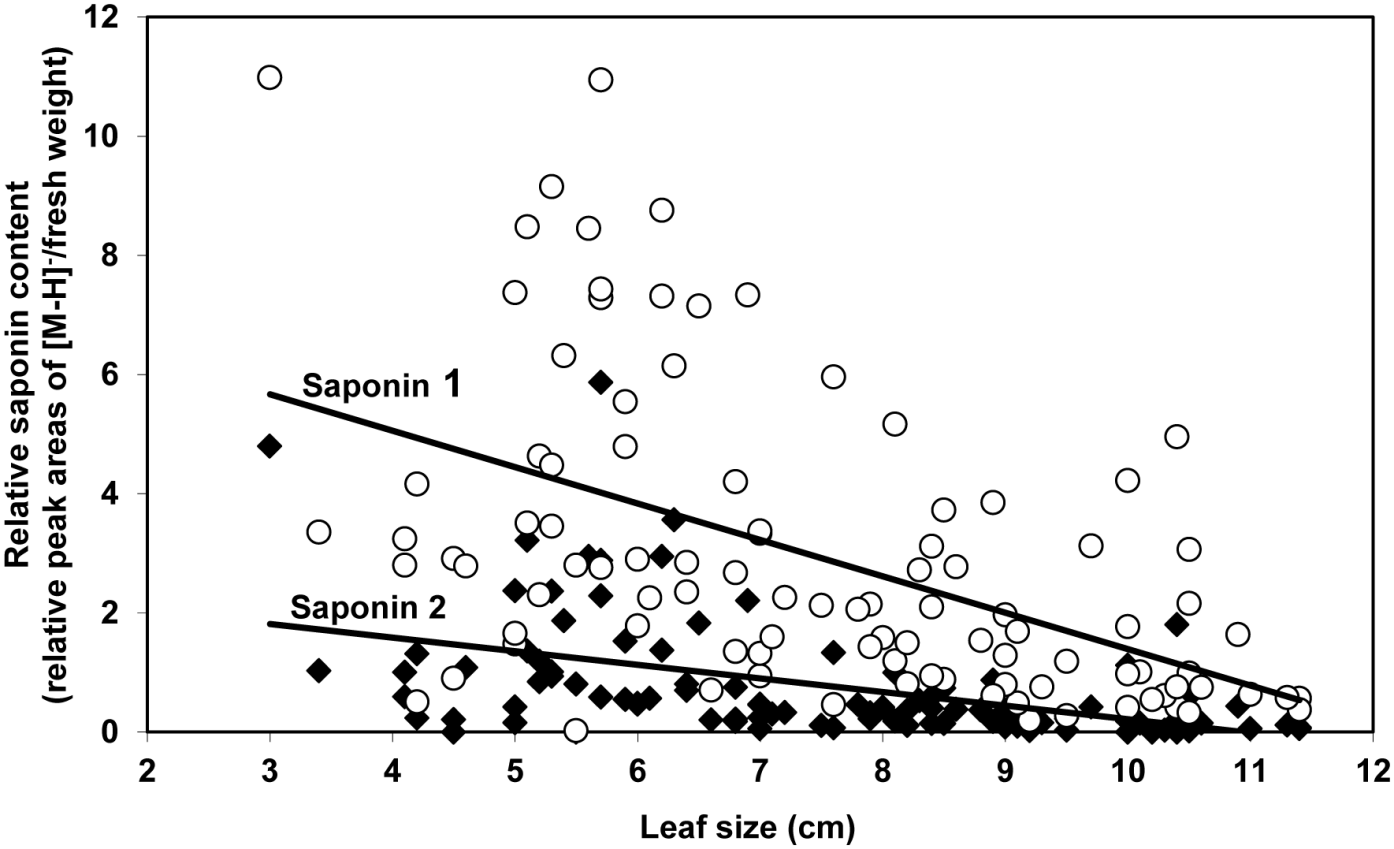
Relative content of 3-*0*-*β*-cellobiosylhederagenin (saponin 1) and 3-*0*-*β*-cellobiosyloleanolic acid (saponin 2) in leaves of different size in G-type *Barbarea vulgaris* var. *arcuata*. Maximum leaf width ranged from 3.0 to 11.4(n = 100). Units of peak areas for the signal of the molecular ion in the negative-ion mass spectrum [M-H]^−^ divided by 100,000/mg of leaf fresh weight.

In G-type *B. vulgaris*, a significant negative relationship was also found between leaf size and content of saponin 1 (*y* = 7.50–0.61*x*; *n* = 100; *r* = 0.51; *F*_1,98_ = 34.89; *P*≤0.001) and saponin 2 (*y* = 2.50–0.23*x*; *n* = 100; *r* = 0.47; *F*_1,98_ = 27.99; *P*≤0.001) (Fig. 2). In these same leaves of different size, there was a significant positive relationship between saponin and glucosinolate content for both saponin 1 (ln (*y*+1) = 1.36+0.26ln (*x*+1); *n* = 100; *r* = 0.21; *F*_1,98_ = 4.56; *P* = 0.035) and saponin 2 (ln (*y*+1) = 1.47+0.43ln (*x*+1); *n* = 100; *r* = 0.25; *F*_1,98_ = 6.44; *P* = 0.013).

When leaves of different sizes were grouped into three groups according to maximum leaf width (large, >50 mm; medium, 20–50 mm; and small, <20 mm), there were significant differences in the content of glucosinolates (*F*_2,108_ = 224.31; *P*≤0.001) and saponins 1 (*F*_2,81_ = 19.36; *P*≤0.001) and 2 (*F*_2,81_ = 9.06; *P*≤0.001) among the three different leaf sizes (Tables 1 and 2). Small and large leaves had, respectively, the highest and the lowest concentrations of both glucosinolates and saponins. For these leaves of different size, there was a significant positive relationship between saponin and glucosinolate content for both saponin 1 (*y* = 2.10+1.63*10^−5^ *x*; *n* = 30; *r* = 0.40; *F*_1,28_ = 5.41; *P* = 0.027) and saponin 2 (*y* = 1.97+1.02*10^−5^ *x*; *n* = 30; *r* = 0.45; *F*_1,28_ = 7.22; *P* = 0.012) in G-type *B. vulgaris*; for saponin 1 (*y* = 1.81+9.91*10^−5^ *x*; *n* = 30; *r* = 0.79; *F*_1,28_ = 54.55; *P*≤0.001) in *B. verna* (the relationship was not statistically significant for saponin 2); and for saponin 1 (*y* = 6.61+1.80*10^−4^ *x*; *n* = 30; *r* = 0.48; *F*_1,28_ = 8.29; *P* = 0.008) in *B. rupicola*, which did not contain any saponin 2.

**Table 1:** Mean ± SE glucosinolates (µmol/g of leaf fresh weight) concentrations in large, medium and small leaves of *B. rupicola*, *B. verna*, and G- and P- type *B. vulgaris* var. *arcuata*.

	Leaf size	Total glucosinolates	R2OH2PE	S2OH2PE	I3M	4MOI3M	2PE
*B. rupicola*	large	1.44±0.10	0.00±0.00	0.00±0.00	0.02±0.00	0.01±0.00	1.40±0.10
*B. rupicola*	medium	9.04±0.86	0.00±0.00	0.00±0.00	0.43±0.04	0.02±0.00	8.59±0.82
*B. rupicola*	small	14.04±0.95	0.00±0.00	0.00±0.00	0.93±0.06	0.03±0.01	13.07±0.91
*B. verna*	large	2.46±0.15	0.00±0.00	0.00±0.00	0.03±0.00	0.01±0.00	2.42±0.15
*B. verna*	medium	5.54±0.66	0.00±0.00	0.00±0.00	0.10±0.02	0.01±0.00	5.43±0.65
*B. verna*	small	13.02±1.07	0.00±0.00	0.00±0.00	0.43±0.04	0.03±0.01	12.57±1.03
G-type *B.vulgaris*	large	0.36±0.05	0.00±0.00	0.31±0.05	0.05±0.01	0.00±0.00	0.00±0.00
G-type *B.vulgaris*	medium	2.83±0.44	0.00±0.00	2.40±0.37	0.42±0.08	0.00±0.00	0.01±0.00
G-type *B.vulgaris*	small	6.09±0.62	0.00±0.00	4.40±0.43	1.34±0.22	0.01±0.00	0.34±0.07
P-type *B.vulgaris*	large	0.74±0.16	0.63±0.16	0.00±0.00	0.10±0.01	0.00±0.00	0.00±0.00
P-type *B.vulgaris*	medium	4.98±0.96	4.53±0.88	0.00±0.00	0.43±0.09	0.00±0.00	0.02±0.00
P-type *B.vulgaris*	small	11.20±0.64	9.92±0.55	0.00±0.00	1.21±0.11	0.01±0.00	0.06±0.01

Abbreviations for glucosinolates are: (*R*)-2-hydroxy-2-phenylethylglucosinolate (R2OH2PE), (*S*)-2-hydroxy-2-phenylethylglucosinolate (S2OH2PE), indol-3-ylmethylglucosinolate (I3M), 4-methoxyindol-3-ylmethylglucosinolate (4MOI3M), and 2-phenylethylglucosinolate (2PE).

Large, medium and small leaves had a maximum leaf width of >50 mm, 20–50 mm, and <20 mm, respectively. For each plant and leaf size n = 10.

**Table 2:** Mean ± SE 3-*0*-*β*-cellobiosylhederagenin (saponin 1) and 3-*0*-*β*-cellobiosyloleanolic acid (saponin 2) in large, medium, and small leaves within the same plant.

	Type of leaf	Saponin 1	Saponin 2
*B. rupicola*	large	(1.1±1.1)*10^2^	0.00±0.00
*B. rupicola*	medium	(7.4±3.3)*10^3^	0.00±0.00
*B. rupicola*	small	(1.9±0.7)*10^4^	0.00±0.00
*B. verna*	large	(1.5±0.3)*10^4^	(4.5±3.2)*10^2^
*B. verna*	medium	(5.3±1.1)*10^4^	(2.1±1.1)*10^3^
*B. verna*	small	(9.0±0.9)*10^4^	0.00±0.00
G-type *B.vulgaris*	large	(1.2±0.3)*10^4^	(4.4±1.4)*10^3^
G-type *B.vulgaris*	medium	(1.2±0.3)*10^5^	(7.4±1.8)*10^4^
G-type *B.vulgaris*	small	(2.0±0.4)*10^5^	(1.1±0.2)*10^5^

Large, medium and small leaves had a maximum leaf width of >50 mm, 20–50 mm, and <20 mm, respectively. For each plant and leaf type n = 10. Saponin concentrations given as peak areas for the signal of the molecular ion in the negative-ion mass spectrum [M-H]^−/^mg of leaf fresh weight.

There were significant differences in the content of glucosinolates in cotyledons and true leaves of plants of G-type *B. vulgaris*, P-type *B. vulgaris*, NAS-type *B. vulgaris*, *B. vulgaris variegata*, *B. rupicola*, and *B. verna* (*F*_1,134_ = 261.79; *P*≤0.001) (Table 3). True leaves contained approximately 2.3, 7.4, 2.9, 3.0, 3.5, and 4.2 times more glucosinolates than cotyledons in G-type *B. vulgaris*, P-type *B. vulgaris*, NAS-type *B. vulgaris*, *B. vulgaris variegata*, *B. rupicola*, and *B. verna*, respectively. Present in higher concentrations in true leaves than in cotyledons were the individual glucosinolates S2OH2PE (*F*_1,134_ = 51.45; *P*≤0.001); R2OH2PE (*F*_1,134_ = 44.22; *P*≤0.001); I3M (*F*_1,134_ = 18.63; *P*≤0.001); and 2-phenylethylglucosinolate (2PE) (*F*_1,134_ = 399.78; *P*≤0.001). Concentrations of S2OH2PE were 2.6 and 3.3 times higher in true leaves than in cotyledons in G-type *B. vulgaris* and *B. vulgaris variegata*, respectively. Concentrations of R2OH2PE were up to 18.8 times higher in true leaves than in cotyledons in P-type *B. vulgaris*. In *B. verna*, concentrations of I3M were up to 4.8 times higher in true leaves than in cotyledons. In G-type *B. vulgaris*, concentrations of 2PE were up to 36.0 times higher in true leaves than in cotyledons. Concentrations of 4MOI3M, however, were lower in true leaves than in cotyledons (*F*_1,134_ = 113.77; *P*≤0.001). In *B. verna*, concentrations of 4MOI3M were up to 24.0 times lower in true leaves than in cotyledons.

**Table 3:** Mean ± SE glucosinolates (µmol/g of leaf fresh weight) concentrations in cotyledons and true leaves in plants of *B. rupicola*, *B. verna*, G- and P-type *B. vulgaris* var. *arcuata*, NAS-type *B. vulgaris*, and *B. vulgaris* var. *variegata*.

	Type of leaf	Total glucosinolates	S2OH2PE	R2OH2PE	I3M	4MOI3M	2PE
G-type *B.vulgaris*	cotyledon	1.439±0.147	1.147±0.120	0.010±0.005	0.262±0.036	0.018±0.004	0.001±0.001
G-type *B.vulgaris*	true leaf	3.320±0.410	3.046±0.384	0.004±0.003	0.231±0.029	0.002±0.001	0.036±0.014
P-type *B.vulgaris*	cotyledon	0.649±0.112	0.000±0.000	0.352±0.071	0.270±0.050	0.014±0.002	0.013±0.006
P-type *B.vulgaris*	true leaf	4.860±0.643	0.000±0.000	4.444±0.620	0.399±0.061	0.001±0.000	0.016±0.005
NAS-type *B.vulgaris*	cotyledon	0.329±0.066	0.000±0.000	0.016±0.002	0.164±0.041	0.008±0.001	0.141±0.028
NAS-type *B.vulgaris*	true leaf	0.956±0.135	0.000±0.000	0.023±0.005	0.181±0.020	0.002±0.001	0.750±0.125
*B. rupicola*	cotyledon	1.985±0.092	0.000±0.000	0.010±0.002	0.066±0.009	0.019±0.002	1.889±0.087
*B. rupicola*	true leaf	7.042±0.415	0.000±0.000	0.012±0.003	0.199±0.017	0.003±0.001	6.827±0.405
*B.vulgaris variegata*	cotyledon	1.052±0.385	0.857±0.336	0.000±0.000	0.147±0.064	0.007±0.002	0.042±0.020
*B.vulgaris variegata*	true leaf	3.188±0.773	2.841±0.676	0.002±0.002	0.256±0.097	0.002±0.000	0.087±0.023
*B. verna*	cotyledon	2.061±0.088	0.000±0.000	0.005±0.002	0.053±0.006	0.024±0.002	1.979±0.083
*B. verna*	true leaf	8.662±0.509	0.000±0.000	0.012±0.003	0.255±0.017	0.001±0.000	8.385±0.501

Abbreviations for glucosinolates are: (*R*)-2-hydroxy-2-phenylethylglucosinolate (R2OH2PE), (*S*)-2-hydroxy-2-phenylethylglucosinolate (S2OH2PE), indol-3-ylmethylglucosinolate (I3M), 4-methoxyindol-3-ylmethylglucosinolate (4MOI3M), and 2-phenylethylglucosinolate (2PE).

For true leaves, the largest true leaf of each plant was taken. For each plant and leaf type, replication was: n = 15 for G- and P-type *B. vulgaris* and *B. rupicola*, n = 13 for NAS-type *B. vulgaris*, n = 10 for *B. verna*, and n = 5 for *B. vulgaris* var. *variegata*.

There were significant differences in the content of saponins 1 (*F*_1,35_ = 32.48; *P*≤0.001) and 2 (*F*_1,35_ = 5.49; *P* = 0.025) in true leaves and cotyledons of plants of G-type *B. vulgaris* and *B. verna* (Table 4). No saponins were found in cotyledons. Similarly, when comparing true leaves and cotyledons of plants of NAS-type *B. vulgaris*, *B. vulgaris variegata*, and *B. rupicola*, we found significant differences in the content of saponins 1 (*F*_1,40_ = 48.54; *P*≤0.001) and 2 (*F*_1,40_ = 1400.14; *P*≤0.001) (Table 4). No saponins were found in true leaves and cotyledons of *B. rupicola*. In NAS-type *B. vulgaris*, saponin 1 was found in all true leaves and in 3 out of 13 cotyledons analyzed (the peak areas of the signal of [M-H]^−^ were on average 9.7 times smaller for cotyledons than for true leaves in the plants which cotyledons contained saponins). In *B. vulgaris variegata*, saponin 1 was found in all true leaves and in 1 out of 5 cotyledons analyzed (the peak areas of the signal of [M-H]^−^ were 7.1 times smaller for cotyledons than for true leaves in the plant of *B. vulgaris variegata* which cotyledon contained saponins). Saponin 2 was not detected in cotyledons. In plants of G- and P-type *B. vulgaris*, *B. rupicola*, and *B. verna*, 5 h after cutting all leaves except either one cotyledon or one true leaf, there were no differences in the content of glucosinolates (*F*_1,64_ = 0.11; *P* = 0.743) (Table S1) and saponins 1 (*F*_1,48_ = 0.42; *P* = 0.436) and 2 (*F*_1,48_ = 0.52; *P* = 0.473) (Table S2) compared to the same type of leaves in intact plants with all their leaves remaining (Table S1). The glucosinolate content of *Barbarea* seeds is shown on Table 5. *Barbarea* seeds did not contain saponins 1 and 2.

**Table 4:** Mean ± SE 3–*0*-*β*-cellobiosylhederagenin (saponin 1) and 3–*0*-*β*-cellobiosyloleanolic acid (saponin 2) in cotyledons and true leaves in plants of *B. rupicola*, *B. verna*, G-type *B. vulgaris* var. *arcuata*, NAS-type *B. vulgaris*, and *B. vulgaris* var. *arcuata*.

	Type of leaf	Saponin 1	Saponin 2
G-type *B.vulgaris*	cotyledon	0.00±0.00	0.00±0.00
G-type *B.vulgaris*	true leaf	0.15±0.04	0.04±0.06
*B. rupicola*	cotyledon	0.00±0.00	0.00±0.00
*B. rupicola*	true leaf	0.00±0.00	0.00±0.00
*B. verna*	cotyledon	0.00±0.00	0.00±0.00
*B. verna*	true leaf	0.05±0.01	0.00±0.00
NAS-type *B.vulgaris*	cotyledon	(1.3±0.8)*10^4^	0.00±0.00
NAS-type *B.vulgaris*	true leaf	(4.0±0.6)*10^5^	(1.2±0.2) *10^5^
*B. vulgaris variegata*	cotyledon	(2.0±2.0)*10^4^	0.00±0.00
*B. vulgaris variegata*	true leaf	(4.9±0.9) *10^5^	(1.3±0.5) *10^5^

Traces of saponin 2 (less than 0.01 µmol/g plant fresh weight) were also detected in *B. verna*.

For true leaves, the largest true leaf of each plant was taken. For each plant and leaf type and treatment: n = 10 for G-type *B.vulgaris* and *B. verna*, n = 13 for NAS-type *B. vulgaris*, and n = 5 for *B. rupicola* and *B. vulgaris variegata*. Saponin concentrations given as µmol/g of leaf fresh weight for G-type *B. vulgaris* var. *arcuata* and *B. verna* and as peak areas for the signal of the molecular ion in the negative-ion mass spectrum [M-H]^−/^mg of leaf fresh weight for *B. rupicola*, NAS-type *B. vulgaris*, and *B. vulgaris* var. *arcuata*. Analyses were also conducted with P-type *B. vulgaris* var. *arcuata*, which did not have any saponins 1 and 2.

**Table 5:** Mean ± SE glucosinolates (µmol/g of seed) in seeds of *B.verna* (n = 4), G-type *B. vulgaris* var. *arcuata* (n = 2), P-type *B. vulgaris* var. *arcuata* (n = 4), and *B. vulgaris* var. *variegata* (n = 2).

Glucosinolates	*B. verna*	*B. vulgaris*G-type	*B. vulgaris*P-type	*B. vulgaris* *variegata*
Total	76.73±3.91	31.65±0.59	60.32±7.94	53.64±0.08
R2OH2PE	0.02±0.01	0.56±0.01	53.51±4.78	1.18±0.01
S2OH2PE	0.00±0.00	30.69±0.53	0.00±0.00	43.21±0.18
I3M	0.09±0.01	0.15±0.01	0.22±0.01	0.18±0.01
2PE	76.62±3.90	0.25±0.04	6.59±3.46	9.08±0.08

Abbreviations for glucosinolates are: (*R*)-2-hydroxy-2-phenylethylglucosinolate (R2OH2PE), (*S*)-2-hydroxy-2-phenylethylglucosinolate (S2OH2PE), indol-3-ylmethylglucosinolate (I3M), and 2-phenylethylglucosinolate (2PE).

Saponins 1 and 2 were not found in the seeds.

**Figure 3:**
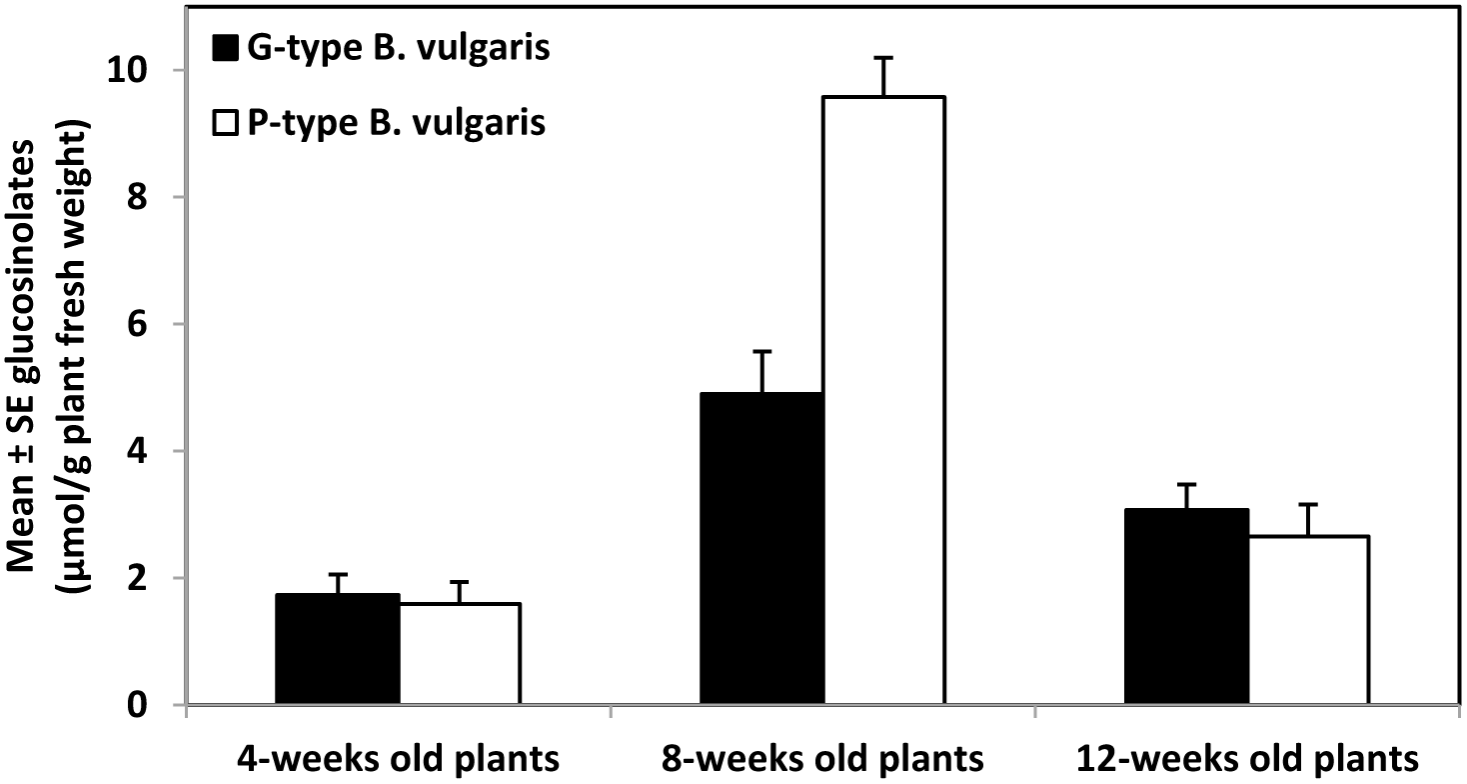
Mean ± SE glucosinolates (µmol/g of plant fresh weight) in the whole foliage of G- and P-type *Barbarea vulgaris* var. *arcuata* plants of 4-, 8-, and 12-weeks old plants (n = 10).

**Figure 4:**
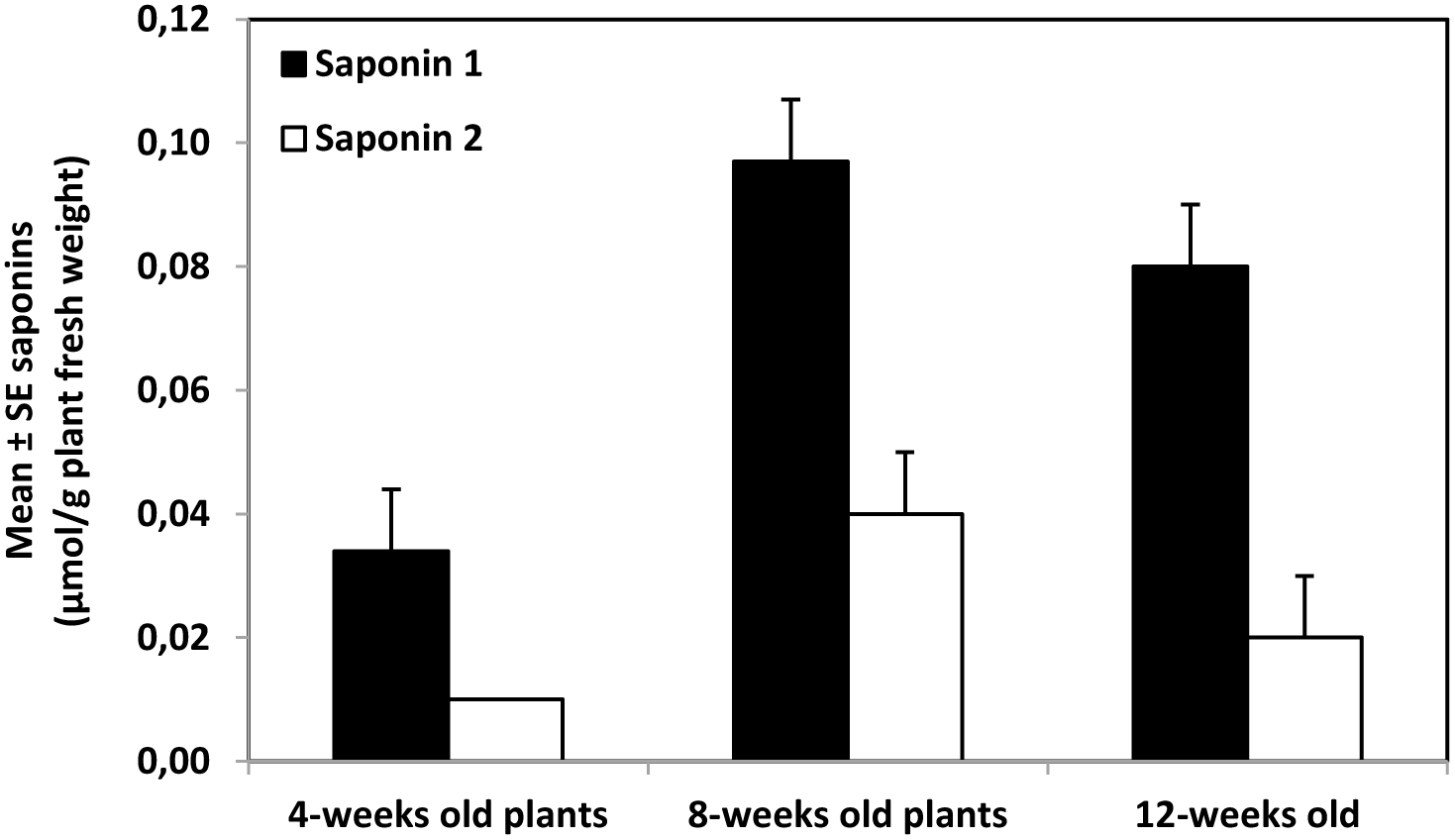
Mean ± SE 3-*0*-*β*-cellobiosylhederagenin (saponin 1) and 3-*0*-*β*-cellobiosyloleanolic acid (saponin 2) in the whole foliage of G-type *Barbarea vulgaris* var. *arcuata* plants of 4-, 8-, and 12-weeks old plants (n = 10).

When analyzing the whole plant foliage, there were significant differences in glucosinolate content among plants of different age in both G-type (*F*_2,27_ = 10.70; *P*≤0.001) and P-type plants (*F*_2,27_ = 56.29; *P*≤0.001) (Fig. 3). In both G- and P-type *B. vulgaris* plants, total glucosinolate content was highest in 8-week-old plants and lowest in 4-week-old plants. Among the individual glucosinolates in G-type *B. vulgaris*, those that varied the most with plant age were I3M (*F*_2,27_ = 43.87; *P*≤0.001), S2OH2PE (*F*_2,27_ = 7.58; *P* = 0.002) and 4MOI3M (*F*_2,27_ = 4.49; *P* = 0.021), while 2PE (*F*_2,27_ = 0.15; *P* = 0.865) and R2OH2PE (*F*_2,27_ = 0.30; *P* = 0.741) did not show significant variation (Table 6). Among the individual glucosinolates that varied the most with plant age in P-type *B. vulgaris* were R2OH2PE (*F*_2,27_ = 66.32; *P*≤0.001), I3M (*F*_2,27_ = 30.78; *P*≤0.001), and 4MOI3M (*F*_2,27_ = 4.63; *P* = 0.018), while 2PE (*F*_2,27_ = 3.33; *P* = 0.051) and S2OH2PE (*F*_2,27_ = 2.14; *P* = 0.138) did not vary significantly (Table 6). There were also significant differences in the content of saponins 1 (*F*_2,27_ = 8.51; *P* = 0.001) and 2 (*F*_2,27_ = 3.86; *P* = 0.034) among the G-type *B. vulgaris* plants of different age (Fig. 4). As in the case of total glucosinolate content, the content of saponins 1 and 2 was highest in 8-week-old plants and lowest in 4-week-old plants. For G-type *B. vulgaris* plants of different ages, there was also a significant positive relationship between saponin and glucosinolate content for both saponin 1 (*y* = 1.14+30.35*x*; *n* = 28; *r* = 0.65; *F*_1,26_ = 19.05; *P*≤0.001) and saponin 2 (*y* = 1.90+62.90*x*; *n* = 28; *r* = 0.68; *F*_1,26_ = 22.12; *P*≤0.001).

**Table 6:** Mean ± SE glucosinolates (µmol/g of plant fresh weight) in foliage of G- and P- type *B. vulgaris* var. *arcuata* plants 4-weeks old, 8-weeks old, and 12-weeks old (n = 10).

Glucosinolates	*B. vulgaris* var. *arcuata*G-type			*B. vulgaris* var. *arcuata*P-type		
	4-weeks old	8-weeks old	12-weeks old	4-weeks old	8-weeks old	12-weeks old
Total	1.73±0.32	4.89±0.67	3.06±0.40	1.58±0.34	9.57±0.62	2.66±0.50
R2OH2PE	0.22±0.11	0.19±0.04	0.14±0.03	1.02±0.24	8.97±0.64	2.45±0.47
S2OH2PE	1.46±0.35	4.20±0.67	2.78±0.37	0.50±0.38	0.00±0.00	0.00±0.00
I3M	0.04±0.01	0.47±0.06	0.13±0.02	0.04±0.01	0.56±0.08	0.18±0.04
4MOI3M	0.00±0.00	0.02±0.01	0.00±0.00	0.00±0.00	0.01±0.00	0.02±0.00
2PE	0.01±0.00	0.01±0.00	0.01±0.00	0.02±0.01	0.03±0.01	0.01±0.00

Abbreviations for glucosinolates are: (*R*)-2-hydroxy-2-phenylethylglucosinolate (R2OH2PE), (*S*)-2-hydroxy-2-phenylethylglucosinolate (S2OH2PE), indol-3-ylmethylglucosinolate (I3M), 4-methoxyindol-3-ylmethylglucosinolate (4MOI3M), and 2-phenylethylglucosinolate (2PE). Traces of 4MOI3M (less than 0.01 µmol g^−1^ of plant fresh weight) were found in 4- and 12-weeks old G-type *B. vulgaris* var. *arcuata* plants and in 4-weeks old P-type *B. vulgaris* var. *arcuata* plants. Traces of S2OH2PE (less than 0.01 µmol g^−1^ of plant fresh weight) were found in 12-weeks old P-type *B. vulgaris* var. *arcuata* plants.

### Oviposition Preference Tests between Leaves of Different Size within the Same Plant

**Figure 5:**
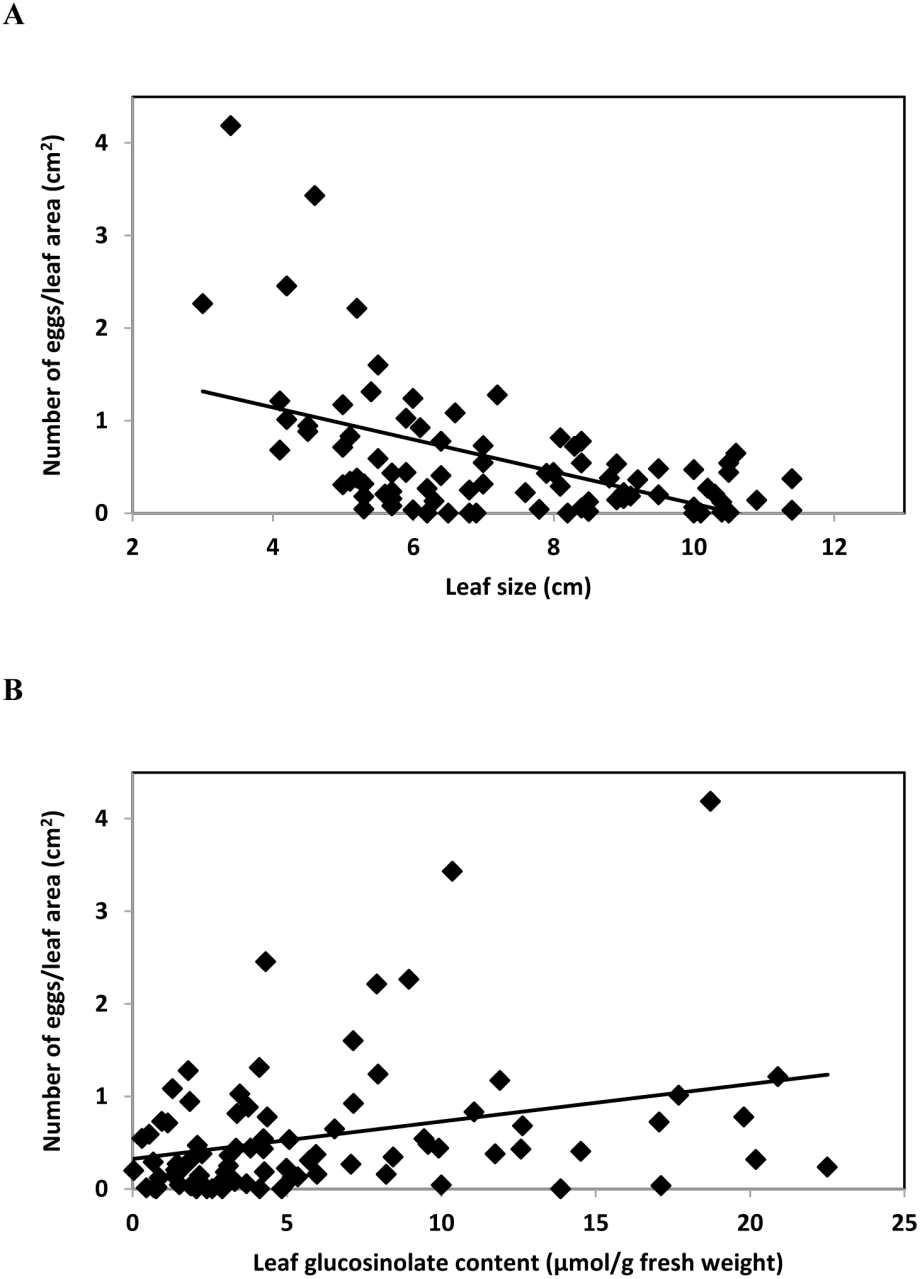
Correlation between leaf size and number of eggs laid by *P. xylostella* per leaf area (A) and between leaf glucosinolate content and number of eggs laid by *P. xylostella* per leaf area (B). Leaves of G-type *Barbarea vulgaris* var. *arcuata* plants were used (n = 84).

There was a significant negative relationship between leaf size and number of eggs laid by *P. xylostella* per leaf area (*y* = 1.84–0.17*x*; *n* = 84; *r* = 0.58; *F*_1,83_ = 42.15; *P*≤0.001) (Fig. 5A). The number of eggs laid per leaf area was also positively correlated with leaf glucosinolate content (*y* = 0.32+0.04*x*; *n* = 84; *r* = 0.34; *F*_1,83_ = 10.40; *P* = 0.002) (Fig. 5B). In the case of *P. xylostella* oviposition preference between true leaves and cotyledons within the same plant, significantly more eggs were laid on true leaves than on cotyledons (*F*_1,18_ = 12.62; *P* = 0.002). When considering the numbers of eggs laid by *P. xylostella* per leaf area, however, these differences were not statistically significant (*F*_1,18_ = 0.47; *P* = 0.502) (Table 7).

**Table 7:** Leaf areas of cotyledons and true leaves of *B. rupicola*, *B. verna*, and G-type *B. vulgaris*, and numbers of eggs laid by *P. xylostella* on cotyledons and true leaves in two-choice preference tests (n = 4 for each plant and leaf type).

	Type of leaf	Leaf area (cm^2^)	Eggs per leaf	Eggs/cm^2^ of leaf area
*B. rupicola*	cotyledon	0.48±0.09	1.25±0.95	2.73±2.29
*B. rupicola*	true leaf	4.70±1.07	9.25±3.15	2.23±0.77
*B. verna*	cotyledon	1.31±0.26	1.75±1.11	1.17±0.53
*B. verna*	true leaf	5.41±1.58	8.75±4.31	2.20±1.02
G-type *B.vulgaris*	cotyledon	0.91±0.14	2.25±1.31	2.76±1.82
G-type *B.vulgaris*	true leaf	5.19±0.80	9.00±4.38	2.48±1.66

Data shown as mean ± SE.

### Survival of Larvae on True Leaves and Cotyledons within the Same Plant

On true leaves of plants, 100% and 80% of *P. xylostella* larvae survived after 5 days on P-type *B. vulgaris* and *B. rupicola*, respectively (for each *Barbarea* tested, n = 5 plants, each with 5 larvae). No larvae survived the 5-day period on true leaves of G-type *B. vulgaris*, *B. vulgaris variegata*, NAS-type *B. vulgaris*, and *B. verna*. On cotyledons, however, survival of *P. xylostella* larvae after 5 days was high for all *Barbarea* plants tested: 100%, 100%, 80%, 100%, 100% and 100% on G-type *B. vulgaris,* P-type *B. vulgaris*, *B. vulgaris variegata*, NAS-type *B. vulgaris*, *B. rupicola*, and *B. verna*, respectively.

## Discussion

Our research shows that in *Barbarea*, the only genus of the Brassicaceae family known to simultaneously contain glucosinolates and saponins, content of these two plant defense compounds are negatively correlated with leaf size. Oviposition preference by *P. xylostella* was also negatively correlated with leaf size because *P. xylostella* laid more eggs per leaf area on smaller leaves than on larger ones. *P. xylostella* and many other herbivores use plant secondary metabolites as “fingerprints” to recognize hosts and oviposit on them [34], [53]. In our study, attraction to glucosinolates seemed to be more important for ovipositing *P. xylostella* than avoidance of saponins, which is consistent with the presence and absence of glucosinolates and saponins, respectively, on the leaf surface of *Barbarea* in concentrations perceivable by *P. xylostella* [34]. In *Barbarea* plants with saponins, these were found in true leaves of all sizes, while no saponins (or very small amounts of them) were found in cotyledons. Larvae of *P. xylostella* could survive on cotyledons, even in those *Barbarea* plants whose true leaves contained enough saponin concentrations to prevent their survival.

In Lepidoptera, survival is greatly determined by the oviposition behavior of adult females, as immature stages have limited mobility [54]. Consequently, most ovipositing Lepidoptera prefer to oviposit on hosts where their larvae are able to survive, but there are cases in which the correlation between oviposition preference and larval performance is poor, and several hypotheses have been put forward to interpret this apparently non-adaptative behavior [55]–[57]. With few exceptions, such as ovipositing on *Barbarea* [28], [39], [40], *P. xylostella* oviposition preference and larval performance are positively correlated [58]. However, the oviposition preference for smaller *Barbarea* leaves over larger ones demonstrated here for *P. xylostella* seems to be a non-adaptive mechanical response to cues given by plant secondary metabolites (glucosinolates) specific from their cruciferous host plants. Given that no *P. xylostella* larvae survive on resistant *Barbarea*, and that survival of larvae is less likely on small leaves that contain high concentrations of saponins, there cannot be any selective advantage in the oviposition behavior of *P. xylostella* on *Barbarea*. The relatively low content of saponins in larger leaves of *Barbarea* would make *P. xylostella* more likely to survive on the plant, yet larger leaves also have relatively low concentrations of glucosinolates, which make them less stimulatory for *P. xylostella* larvae [35]. The preference of *P. xylostella* moths for younger leaves within a *Barbarea* plant represents a second “oviposition mistake”, on top of the known “oviposition mistake” of *P. xylostella* preferring resistant *Barbarea* plants over other host plants that allow survival of its larvae [28], [40]–[42].

Cotyledons serve as a storage of nutrients for the growing plant and they are the first photosynthetic tissue appearing above-ground when germination occurs [59]. Cotyledons of brassicaceous plants usually contain variable amounts of glucosinolates [8], [60]. In *Barbarea* plants, glucosinolates, which can defend the plants against generalist herbivores, were present in cotyledons, but saponins, which could also protect the plant against specialist herbivores like *P. xylostella*, were not (or were present in very low concentrations). No saponin 2 was detected in cotyledons, indicating that synthesis of saponin 2 could be subsequent to that of saponin 1 (assuming that saponins are not translocated from cotyledons to other parts of the plant). Unlike glucosinolates, saponins were not found in the seeds of *Barbarea*, indicating that saponins may start being produced in cotyledons and true leaves after some time once true leaves appear. Lack of saponins in seeds and cotyledons indicates that, for some time, at the seedling stage, the plant may not be protected against *P. xylostella* and other herbivores. However, given the small size of cotyledons, they do not provide sufficient food for a *P. xylostella* larva to develop from first instar to pupa (Badenes-Perez, personal observation). Even though *P. xylostella* is known to oviposit on cotyledons of crucifer seedlings in the field, upon egg hatch, larvae move to true leaves, where they prefer to feed [61]. With the exception of 4MOI3M, we found that cotyledons contained lower concentrations of glucosinolates than true leaves in the same plant. This, together with the low frequency of cotyledons with saponins, the low concentrations of saponins found in those cotyledons with saponins, and the ensuing survival of *P. xylostella* larvae on cotyledons, indicates that cotyledons are not as protected from herbivory as true leaves. However, as *Barbarea* spp. are early successional biennial plants that appear early in the season [62], cotyledons might be important for the plant only for a relatively short time, when the presence of herbivores and the visibility of the plant as a seedling may be relatively low. This would also be in agreement with the plant apparency hypothesis [63].

Our analyses show that total glucosinolate content in 4-, 8-, and 12-week-old plants varies, but not in the same linear manner as oviposition preference by *P. xylostella* varies among plants of similar ages as described by Badenes-Perez et al (2005). Non-linear ontogenetic changes in the content of defense compounds have been interpreted as part of a dynamic pattern, also affected by the development of herbivore tolerance and resource allocation constraints in the plant [59]. Besides plant glucosinolate content, the increase in leaf area and leaf number that occurs with plant age may affect oviposition preference by *P. xylostella* [52]. In *B. vulgaris*, the increase in number of leaves and total leaf area when comparing 6- and 12-week-old plants was 11.6 and 42.2 fold, respectively [52].

The simultaneous presence of high content of glucosinolates and saponins in small/young leaves of *Barbarea*, which are the most valuable for the plant, but also the most attractive to ovipositing *P. xylostella*, provides protection against this specialist herbivore. The association between glucosinolates and saponins could indicate that, from an evolutionary point of view, in *Barbarea*, saponins might have appeared after glucosinolates, enabling plants to be defended against insects that had adapted to glucosinolate-defended plants. Saponins would then be what has been called a “second line of defense”, appearing as a response to herbivores that have overcome the “first line of defense” provided by glucosinolates [25], [64].

## Materials and Methods

### Ethics Statement

Insects collected in Kenya were collected at Athi River, 40 km southeast of Nairobi, Kenya, in 2005 by Dr. Bernhard Löhr, and sent by him in July 2005 to MPICE in Jena under EU permit number EG-D-TH1-390390 AG39/2005. Insects collected in Spain were collected in 2013 in Arganda del Rey, Madrid, at the experimental farm “La Poveda”, which belongs to the Institute of Agricultural Sciences (CSIC). A permit was not required for the collection of insects at the collecting site in Spain.

### Plant Growth and Insect Culture

Experiments were conducted in the laboratory at the Max Planck Institute for Chemical Ecology in Jena, Germany. *Barbarea rupicola* Moris, *B. verna* (Mill.) Asch., and *B. vulgaris* var. *variegata* seeds were purchased from B & T World Seeds (Aigues-Vives, France). *Barbarea vulgaris* var. *arcuata* G-type seeds were purchased from Rieger-Hofmann GmbH (Blaufelden-Raboldshausen, Germany) and P-type seeds were collected in Tissø (Denmark) and donated to us by Dr. Jens K. Nielsen. Seeds of NAS-type *B. vulgaris* were collected in The Netherlands and donated to us by Dr. Hanneke van Leur. The NAS-type of *B. vulgaris* was not classified varietally, although morphologically they would belong to what botanists consider var. *arcuata* or var. *vulgaris* [65]. Additional G-type *Barbarea vulgaris* var. *arcuata* seeds from Jena (Germany) were provided by Dr. Tamara Krügel. All plants used in the experiments were grown in pots in the greenhouse using a substrate of peat moss with clay. In the experiments testing the effect of different leaf size on glucosinolate and saponin content, plants were approximately 10 weeks old at the time when the experiments were conducted and they were grown in 20-cm-diameter pots. To compare differences in glucosinolate and saponin content between true leaves and cotyledons, 4-week-old plants were used and they were grown in 8-cm-diameter pots. Glucosinolate and saponin content was also compared among plants that were 4, 8, and 12 weeks old and grown in 15-cm-diameter pots. All plants used in the experiments were grown in the glasshouse at 22–28°C under 16 h supplemental light from Master Sun-T PIA Agro 400 or Master Sun-T PIA Plus 600 W Na lights (Philips, Turnhout, Belgium). *P. xylostella* used in the experiments were either collected in Kenya (provided by Dr. Bernhard Löhr) or collected in Spain. Insects were later reared on cabbage plants in a climate-controlled chamber (16∶8 h light:dark, 21±2°C and 55±5 RH).

### Analysis of Glucosinolates and Saponins in Barbarea spp

Glucosinolate and saponin content in individual cotyledons, in individual true leaves, in foliage of whole plants, and in seeds was determined as in Badenes-Perez et al. (2010). Cotyledons and leaves were cut approximately from the middle of their petiole. In the experiment comparing cotyledons and true leaves, the first (largest) true leaf in 4-week-old plants was used in the analyses. Foliage of whole plants was harvested by cutting approximately half of the plant from the crown in the case of the comparison of foliage among plants 4, 8, and 12 weeks old. In the experiment testing whether changes in glucosinolate and saponin content occurred when only one cotyledon or one true leaf was left per plant, all the leaves of the plant, except either one cotyledon or the largest true leaf, were cut with scissors. The content of glucosinolates and saponins in the remaining cotyledon or true leaf was compared to a control of similar leaves in intact plants (having no leaves cut) 5 hours after removal of leaves. This experiment was arranged to assess whether an associated experiment conducted to test *P. xylostella* oviposition preference between true leaves and cotyledons could be influenced by glucosinolate and saponin induction at the time as a result of the mechanical removal of leaves. The time period of 5 hours to study changes in glucosinolate and saponin content in the plants was set to coincide with the time during which *P. xylostella* had laid most of its eggs in the course of the experiment [31], [66]. For glucosinolate and saponin analysis in *Barbarea* seeds, 20 mg of seeds were analyzed for each plant type and replicate. Glucosinolates and saponins were extracted with 80% aqueous methanol (methanol:water 80∶20, v:v). For glucosinolate determination, 4-hydroxybenzylglucosinolate was added as an internal standard. The methanolic extract was loaded onto DEAE Sephadex columns, followed by washing steps and by sulfatase treatment and elution of desulfoglucosinolates. Desulfoglucosinolates were separated on reversed-phase chromatography and quantified with a diode array detector at 229 nm (Agilent 1100 HPLC system, Agilent Technologies, Waldbronn, Germany), using a relative response factor of 2.0 for aliphatic and 0.5 for indole glucosinolates. The response factors we used were based on Brown et al. (2002). For saponin determination, the HPLC system indicated above was coupled to an ESI ion-trap mass spectrometer (Esquire 6000, Bruker Daltonics, Bremen, Germany) operated in negative mode in the range m/z 250–1700, with skimmer voltage, −40 eV; capillary exit voltage, −150.6 eV; capillary voltage, 4,000 V; nebulizer pressure, 35 psi; drying gas, 10 l min-1; and gas temperature, 330°C. Saponins were quantified by the peak areas for the signal of the molecular ion in the negative-ion mass spectrum [M-H]^−^ and in some of the analyses we used a standard curve created with an isolated standard of saponin 2.

### Oviposition Preference Tests between Leaves of Different Size within the Same Plant

Oviposition preference between leaves of different type within the same plant was assessed in plexiglass tubes 3.0 cm (inner diameter) by 10.0 cm (length) with plants of G-type *B. vulgaris* var. *arcuata*. Each tube had a 0.5-cm-diameter hole in the middle, through which a piece of dental wick soaked with a 10% sugar solution was inserted into the tube as a food source for the moth inside. One mated female moth was placed in each tube, where it was offered two 7.1 cm^2^ circular disks of the abaxial side of *B. vulgaris* leaves. For each tube, the ends of a single tube were attached to two different leaves in the same *B. vulgaris* plant with the help of rubber bands and parafilm. The leaves compared had a difference in maximum leaf diameter ranging from 0 to 58 mm. A total of 42 comparisons involving 84 leaves were conducted (besides these 84 leaves, 16 additional leaves in which *P. xylostella* had not laid any eggs were taken to have more data points to analyze the relationship between leaf size and glucosinolate and saponin content). After one day, the number of eggs on each plant was counted in the laboratory using a dissecting microscope. The leaves used in the oviposition preference experiments were photographed with a digital camera and leaf areas were determined using WinFOLIA leaf area analysis software (Regent Instruments Inc., Quebec, Canada).

Oviposition preference experiments were also conducted with cotyledons and true leaves of the same plant for which all other leaves had been removed by cutting them with scissors. Immediately after cutting the leaves, oviposition preference tests were conducted in 32.5×32.5×32.5 cm polyester cages with 96×26 mesh (MegaView Science Education Services Co., Ltd., Taichung, Taiwan). Multiple cages were used, each of which was considered a replicate. One mated female moth was released in each experimental arena containing one *Barbarea* plant with only one true leaf (the largest) and one cotyledon. The experiment was replicated four times for each comparison. A small plastic cup with a 10% sugar solution on cotton was placed in the middle of the cage to provide a food source for the moths. Moths were allowed to oviposit overnight in the darkness from 19∶00 to 7∶00 h. *P. xylostella* lays most of its eggs during the first 3 h of scotophase and the peak oviposition occurs between 19∶00 and 20∶00 h [31], [66]. The number of eggs on each plant was counted in the laboratory using a dissecting microscope.

### Survival of Larvae on True Leaves and Cotyledons within the Same Plant

Survival of first-instar larvae of *P. xylostella* was monitored over a period of 5 days. Using a brush, one *P. xylostella* larva was placed individually on a plant containing either one true leaf or one cotyledon (five plants and five larvae were used in total per treatment). The plants tested were *B. rupicola*, *B. verna*, G- and P-type *B. vulgaris* var. *arcuata*, *B. vulgaris* var. *variegata,* and NAS-type *B. vulgaris*.

### 4.4. Statistical Analysis

Differences in eggs laid by *P. xylostella* per leaf area and in glucosinolate and saponin content among *Barbarea* leaves of different size were analyzed using analysis of variance (ANOVA) and simple regressions with SPSS. When significant treatment differences were indicated by a significant *F*-test at *P*≤0.05, means were separated by Fisher’s Protected least significant difference (LSD). Differences in *P. xylostella* oviposition preference between cotyledons and true leaves were analyzed with a paired t-test with SPSS. In order to normalize the residuals, data were transformed prior to analysis using a natural log (x+1) function. Although all tests of significance were based on the transformed data, only untransformed data are presented.

## Supporting Information

Table S1 **Mean ± SE glucosinolates (µmol/g of leaf fresh weight) concentrations in cotyledons and true leaves in** ***Barbarea*** **plants six hours after removing the rest of the leaves in the plant or leaving them intact.** As true leaf, the largest true leaf of the plant was taken. For each plant and leaf type and treatment n = 5.
(PDF)

Table S2 **Mean ± SE 3-** ***0*** **-** ***β*** **-cellobiosylhederagenin (saponin 1) and 3-** ***0*** **-** ***β*** **-cellobiosyloleanolic acid (saponin 2) in cotyledons and true leaves of** ***Barbarea*** **plants six hours after removing the rest of the leaves in the plant or leaving them intact.** As true leaf, the largest true leaf of the plant was taken. For each plant and leaf type and treatment n = 5. Saponin concentrations given as µmol/g of leaf fresh weight.
(PDF)
