# Supplementary material for: Insect Attraction versus Plant Defense: Young Leaves High in Glucosinolates Stimulate Oviposition by a Specialist Herbivore despite Poor Larval Survival due to High Saponin Content
Source: PLoS One. 2014 Apr 21;9(4):e95766. doi: 10.1371/journal.pone.0095766 (PMC3994119; doi:10.1371/journal.pone.0095766)
Supplement: Table S1 — Mean ± SE glucosinolates (µmol/g of leaf fresh weight) concentrations in cotyledons and true leaves in Barbarea plants six hours after removing the rest of the leaves in the plant or leaving them intact. As true leaf, the largest true leaf of the plant was taken. For each plant and leaf type and treatment n = 5. (PDF) [file pone.0095766.s001.pdf]

Table S1. Mean ± SE glucosinolates (μmol/g of leaf fresh weight) concentrations in cotyledons and true leaves in *Barbarea* plants 5 hours after removing the rest of the leaves in the plant or leaving them intact. As true leaf, the largest true leaf of the plant was taken. For each plant and leaf type and treatment n=5.

	Type of leaf	Other leaves in the plant	Total glucosinolates	R2OH2PE	S2OH2PE	I3M	4MOI3M	2PE
G-type *B.vulgaris*	cotyledon	removed	1.21 ± 0.13	0.00 ± 0.00	0.97 ± 0.11	0.22 ± 0.05	0.02 ± 0.01	0.00 ± 0.00
G-type *B.vulgaris*	cotyledon	intact	1.54 ± 0.32	0.00 ± 0.00	1.28 ± 0.27	0.24 ± 0.06	0.02 ± 0.01	0.00 ± 0.00
G-type *B.vulgaris*	true leaf	removed	3.19 ± 0.80	0.00 ± 0.00	2.86 ± 0.71	0.27 ± 0.07	0.00 ± 0.00	0.06 ± 0.03
G-type *B.vulgaris*	true leaf	intact	3.69 ± 0.49	0.00 ± 0.00	3.46 ± 0.45	0.19 ± 0.03	0.00 ± 0.00	0.04 ± 0.03
P-type *B.vulgaris*	cotyledon	removed	0.55 ± 0.12	0.25 ± 0.08	0.00 ± 0.00	0.28 ± 0.06	0.02 ± 0.00	0.00 ± 0.00
P-type *B.vulgaris*	cotyledon	intact	0.37 ± 0.06	0.19 ± 0.04	0.00 ± 0.00	0.17± 0.04	0.01 ± 0.01	0.00 ± 0.00
P-type *B.vulgaris*	true leaf	removed	5.18 ± 0.77	4.62 ± 0.68	0.00 ± 0.00	0.55 ± 0.11	0.00 ± 0.00	0.01 ± 0.01
P-type *B.vulgaris*	true leaf	intact	4.11 ± 0.97	3.67 ± 0.91	0.00 ± 0.00	0.42 ± 0.11	0.00 ± 0.00	0.02 ± 0.01
B. rupicola	cotyledon	removed	1.86 ± 0.13	0.01 ± 0.00	0.00 ± 0.00	0.07 ± 0.02	0.02 ± 0.00	1.76 ± 0.12
B. rupicola	cotyledon	intact	1.91 ± 0.16	0.01 ± 0.01	0.00 ± 0.00	0.06 ± 0.02	0.03 ± 0.00	1.82 ± 0.14
B. rupicola	true leaf	removed	6.73 ± 0.49	0.02 ± 0.01	0.00 ± 0.00	0.18 ± 0.03	0.00 ± 0.00	6.54 ± 0.50
B. rupicola	true leaf	intact	8.27 ± 0.87	0.01 ± 0.00	0.00 ± 0.00	0.25 ± 0.03	0.00 ± 0.00	8.01 ± 0.85
B. verna	cotyledon	removed	2.07 ± 0.12	0.01 ± 0.00	0.00 ± 0.00	0.06 ± 0.01	0.02 ± 0.00	1.98 ± 0.11
B. verna	cotyledon	intact	2.05 ± 0.14	0.00 ± 0.00	0.00 ± 0.00	0.04 ± 0.01	0.02 ± 0.00	1.98 ± 0.13
B. verna	true leaf	removed	8.38 ± 0.85	0.01 ± 0.00	0.00 ± 0.00	0.26 ± 0.03	0.00 ± 0.00	8.01 ± 0.83
B. verna	true leaf	intact	8.95 ± 0.63	0.02 ± 0.00	0.00 ± 0.00	0.25 ± 0.02	0.00 ± 0.00	8.68 ± 0.63

Abbreviations for glucosinolates are: (*R*)-2-hydroxy-2-phenylethylglucosinolate (R2OH2PE), (*S*)-2-hydroxy-2-phenylethylglucosinolate (S2OH2PE), indol-3-ylmethylglucosinolate (I3M), 4-methoxyindol-3-ylmethylglucosinolate (4MOI3M), and 2-phenylethylglucosinolate (2PE).
