# Supplementary material for: Insect Attraction versus Plant Defense: Young Leaves High in Glucosinolates Stimulate Oviposition by a Specialist Herbivore despite Poor Larval Survival due to High Saponin Content
Source: PLoS One. 2014 Apr 21;9(4):e95766. doi: 10.1371/journal.pone.0095766 (PMC3994119; doi:10.1371/journal.pone.0095766)
Supplement: Table S2 — Mean ± SE 3- 0 - β -cellobiosylhederagenin (saponin 1) and 3- 0 - β -cellobiosyloleanolic acid (saponin 2) in cotyledons and true leaves of Barbarea plants six hours after removing the rest of the leaves in the plant or leaving them intact. As true leaf, the largest true leaf of the plant was taken. For each plant and leaf type and treatment n = 5. Saponin concentrations given as µmol/g of leaf fresh weight. (PDF) [file pone.0095766.s002.pdf]

Table S2. Mean ± SE 3-*0*-*β*-cellobiosylhederagenin (saponin 1) and 3-*0*-*β*-cellobiosyloleanolic acid (saponin 2) in cotyledons and true leaves of *Barbarea* plants six hours after removing the rest of the leaves in the plant or leaving them intact. As true leaf, the largest true leaf of the plant was taken. For each plant and leaf type and treatment n=5. Saponin concentrations given as µmol/g of leaf fresh weight.

	Type of leaf	Other leaves in the plant	Saponin 1	Saponin 2
B. rupicola	cotyledon	removed	0.00 ± 0.00	0.00 ± 0.00
B. rupicola	cotyledon	intact	0.00 ± 0.00	0.00 ± 0.00
B. rupicola	true leaf	removed	0.04 ± 0.01	0.00 ± 0.00
B. rupicola	true leaf	intact	0.04 ± 0.01	0.00 ± 0.00
B. verna	cotyledon	removed	0.00 ± 0.00	0.00 ± 0.00
B. verna	cotyledon	intact	0.00 ± 0.00	0.00 ± 0.00
B. verna	true leaf	removed	0.05 ± 0.01	0.00 ± 0.00
B. verna	true leaf	intact	0.05 ± 0.02	0.00 ± 0.00
G-type *B.vulgaris*	cotyledon	removed	0.00 ± 0.00	0.00 ± 0.00
G-type *B.vulgaris*	cotyledon	intact	0.00 ± 0.00	0.00 ± 0.00
G-type *B.vulgaris*	true leaf	removed	0.12 ± 0.05	0.03 ± 0.02
G-type *B.vulgaris*	true leaf	intact	0.17 ± 0.06	0.05 ± 0.03
